# Editor's note on: Reef fishes can recognize bleached habitat during settlement: sea anemone bleaching alters anemonefish host selection

**DOI:** 10.1098/rspb.2023.0116

**Published:** 2023-02-08

**Authors:** Proceedings B Editorial team

*Proc. R. Soc. B* **283**, 20152694. (Published online 25 May 2016). (https://doi.org/10.1098/rspb.2015.2694)

Concerns have been raised to both the Editor-in-Chief of the journal and in the public domain about the article ‘Reef fishes can recognize bleached habitat during settlement: sea anemone bleaching alters anemonefish host selection’ by Anna Scott and Danielle L. Dixson, *Proc. R. Soc. B* **283**: 20152694 (published 25 May 2016). In response, the *Proceedings B* Editorial board has recently concluded a detailed two-stage investigation of this article to evaluate whether the issues raised constitute a sufficient basis for the article to be retracted.

In such cases, the journal always looks for clear evidence that an article is seriously flawed or contains clear evidence of misconduct before retracting. In the absence of either and where the situation is less clear cut, the benefit of any doubt is given to the author, leaving readers to decide for themselves. Hence, our aim was to assess the combined weight of the evidence. Furthermore, the investigation focused solely on this article and did not consider issues raised regarding other publications by the same author(s).

In response to the first stage of this investigation, Scott and Dixson published a correction [1] in which they addressed issues raised regarding the study design, including its timeline and the large effect sizes of their treatments. Furthermore, they provided the data underlying the analyses in the paper, which until then had not been publicly available. This correction, and the publication of the data in particular, raised a second set of issues. These were again assessed by a group of *Proceedings B* editors, who also sought outside advice from two independent experts familiar with flume experiments. They were asked to comment on the feasibility of the study as described in Scott and Dixson 2016, considering the correction/clarification provided in [1].

This second stage of the investigation concluded that there is a lack of detail regarding some aspects of the methods, and an absence of notebooks, video recordings or other forms of documentation. However, there is insufficient evidence in support of claims that it would have been practically impossible to collect these data following the study protocols as described.

Furthermore, a quantitative assessment by the editors of the claims that specific patterns in the data are the result of data fabrication/manipulation concluded that these patterns (e.g. the high similarity among some trials and the high frequency of specific values) are in line with expectations given the strong preferences observed, or that a biological explanation cannot be ruled out (e.g. variation among individuals being smaller than expected under binomial sampling). Finally, some issues with the data are more likely the result of mistakes or poor data curation, and their correction would not change the conclusions. Again, it was therefore concluded that the evidence in support of claims that these data have been fabricated/manipulated, and hence are unreliable, are too weak to warrant retraction of the paper.

It should be noted that the data provided allows for replication of the full results reported in the article, and thereby (belatedly) fulfills *Proceedings B*'s open data requirement. However, the raw data, i.e. the location of each fish in the flume at each time point, are not available. While this precludes further investigation of some of the patterns, this does not constitute either evidence in support of misconduct or a breach of the conditions for publication in *Proceedings B*.
